# Supplementary figures and images for: SARS-CoV-2 multi-antigen protein microarray for detailed characterization of antibody responses in COVID-19 patients
Source: PLoS One. 2023 Feb 9;18(2):e0276829. doi: 10.1371/journal.pone.0276829 (PMC9910743; doi:10.1371/journal.pone.0276829)

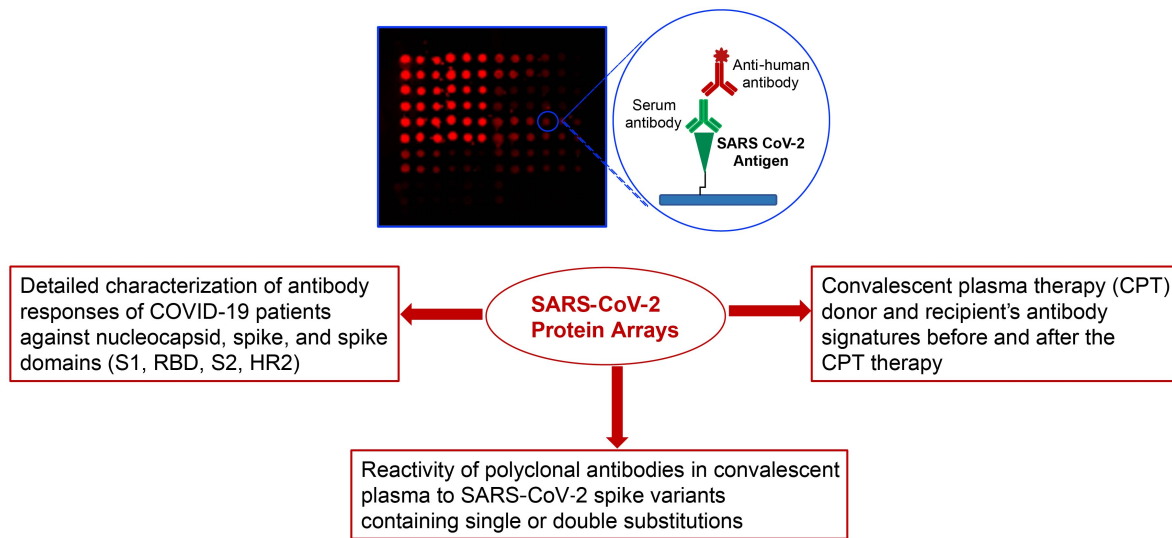

**S1 Fig. Study design for SARS-CoV-2 multi-antigen protein array.**

Supplement: S1 Fig — (PDF) [file pone.0276829.s001.pdf]
