# Supplementary material for: SARS-CoV-2 multi-antigen protein microarray for detailed characterization of antibody responses in COVID-19 patients
Source: PLoS One. 2023 Feb 9;18(2):e0276829. doi: 10.1371/journal.pone.0276829 (PMC9910743; doi:10.1371/journal.pone.0276829)
Supplement: S2 Fig — Sera with highest antibody responses are highlighted with blue frame for each target antigen for both assays. (A) IgG responses to N, S2 domain, RBD and HR2 subdomains determined from protein array for eight convalescent sera along with negative and positive controls. Positive control is a convalescent plasma sample with high S antibody levels determined with ELISA. Signal intensities from duplicates of each target antigen are averaged and normalized to corresponding concentration of human IgG isotype control. (B) IgG responses to N, S2, RBD and HR2 in the same eight sera are detected with ELISA in eleven three-fold serial dilutions starting from 1/100. Absorbance at 450nm is averaged for duplicates and represented using non-linear regression model. (PDF) [file pone.0276829.s002.pdf]

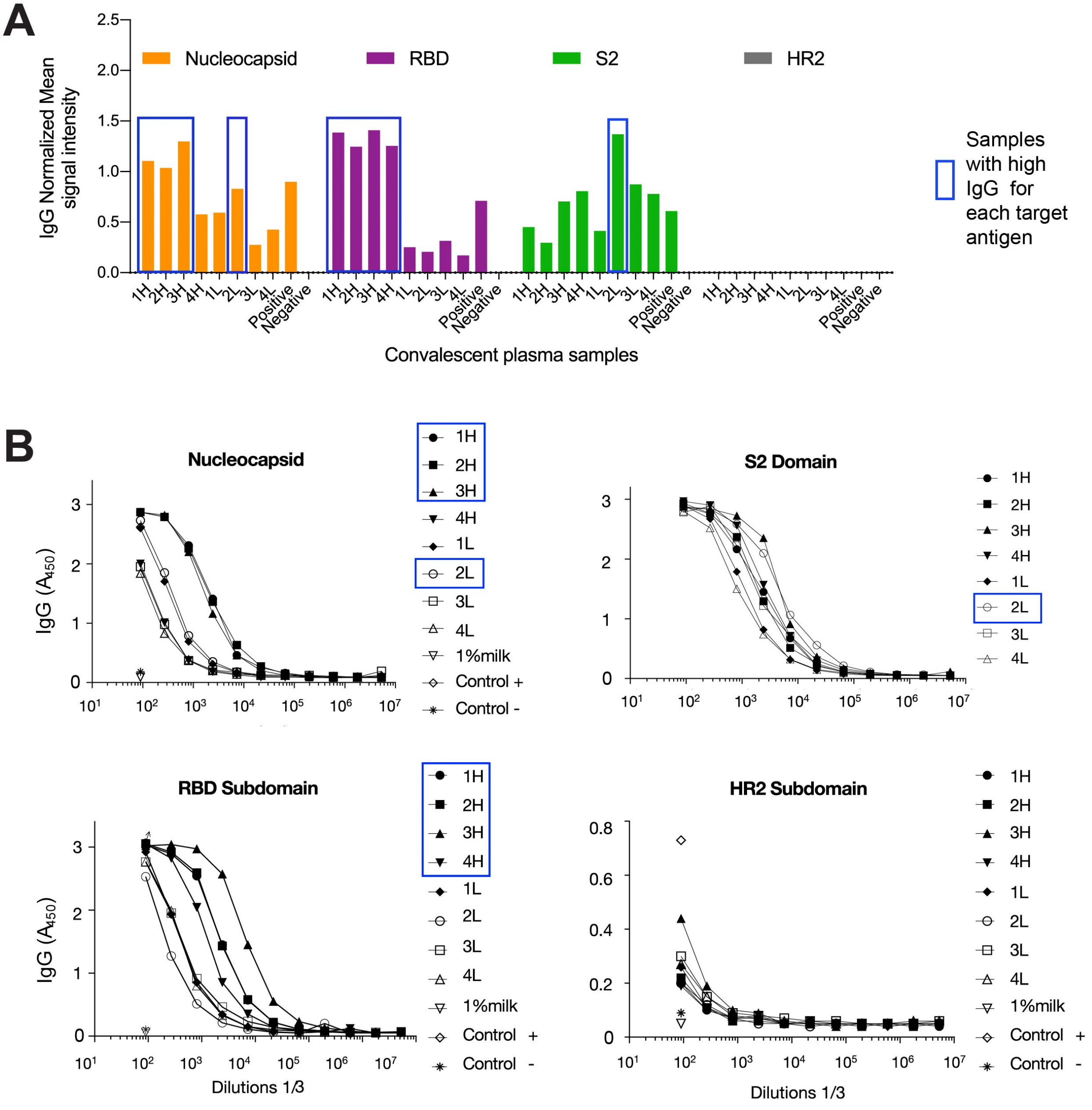

S2 Fig. IgG responses to nucleocapsid protein (N) and S domains detected with protein microarray and ELISA. Sera with highest antibody responses are highlighted with blue frame for each target antigen for both assays. (A) IgG responses to N, S2 domain, RBD and HR2 subdomains determined from protein array for eight convalescent sera along with negative and positive controls. Positive control is a

convalescent plasma sample with high S antibody levels determined with ELISA. Signal intensities from duplicates of each target antigen are averaged and normalized to corresponding concentration of human IgG isotype control. (B) IgG responses to N, S2, RBD and HR2 in the same eight sera are detected with ELISA in eleven three-fold serial dilutions starting from 1/100. Absorbance at 450nm is averaged for duplicates and represented using non-linear regression model.
