# Supplementary material for: SARS-CoV-2 multi-antigen protein microarray for detailed characterization of antibody responses in COVID-19 patients
Source: PLoS One. 2023 Feb 9;18(2):e0276829. doi: 10.1371/journal.pone.0276829 (PMC9910743; doi:10.1371/journal.pone.0276829)
Supplement: S3 Fig — (A) Bar plots are showing IgG, IgM, and IgA response to 4.8 fmol of each S, S2, S1, RBD and N antigen for 18 patients who were infected early in the pandemic. The signal intensities are normalized relative to corresponding concentration of Ig isotype controls and mean values are calculated for the replicates of target antigen for each plasma sample. (B) The ratios of S, S2 and S1 to RBD domain are shown as bar plots for IgG. (PDF) [file pone.0276829.s003.pdf]

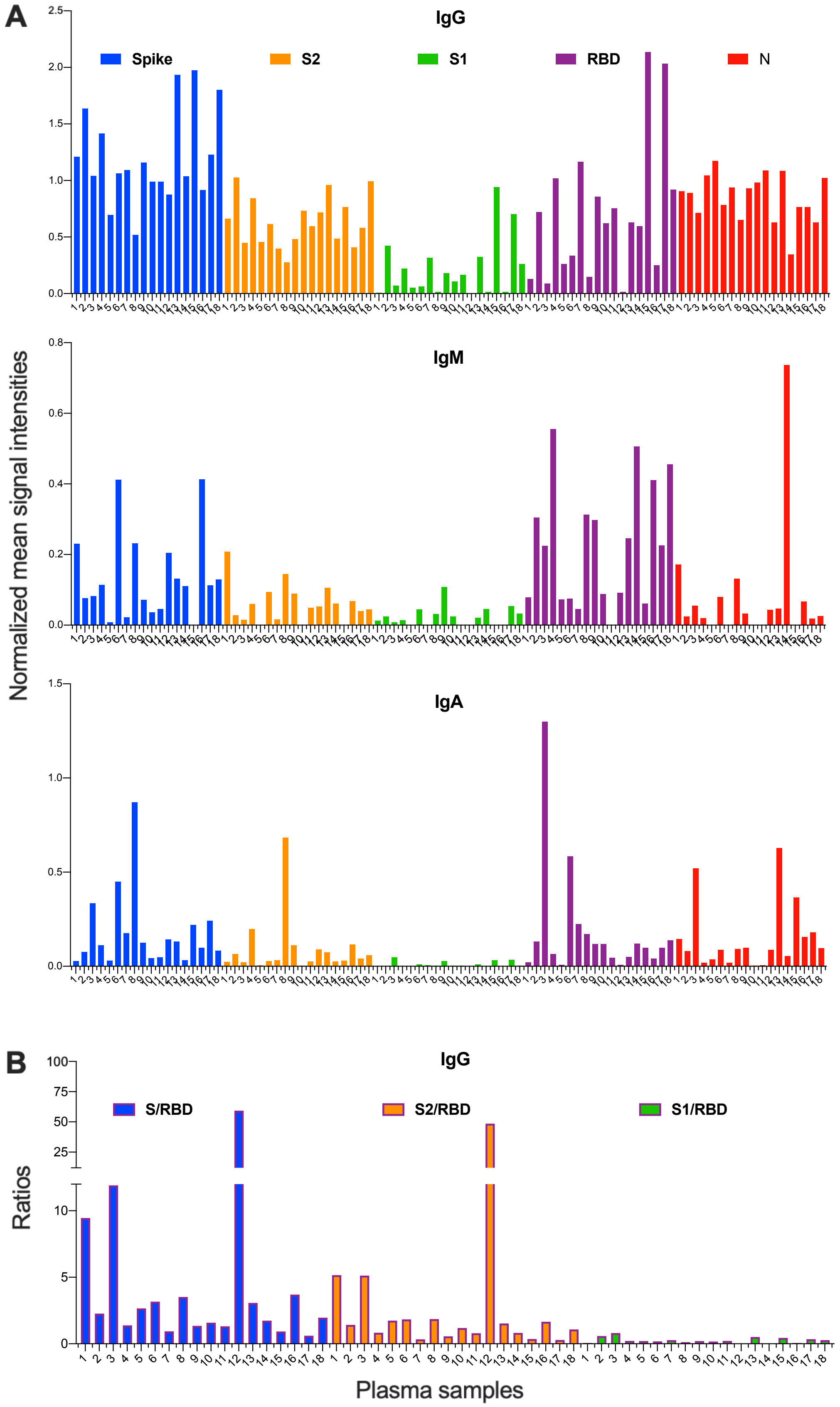
A
IgG
Spike
S2
S1
RBD
N
IgM
IgA
Normalized mean signal intensities
B
IgG
S/RBD
S2/RBD
S1/RBD
Ratios
Plasma samples

S3 Fig. Immunoprofiling COVID-19 patients against multiple SARS-CoV-2 antigens. (A) Bar plots are showing IgG, IgM, and IgA response to 4.8 fmol of each S, S2, S1, RBD and N antigen for 18 patients who were infected early in the pandemic. The signal intensities are normalized relative to corresponding concentration of Ig isotype controls and mean values are calculated for the replicates of target antigen for each plasma sample. (B) The ratios of S, S2 and S1 to RBD domain are shown as bar plots for IgG.
