# Supplementary material for: SARS-CoV-2 multi-antigen protein microarray for detailed characterization of antibody responses in COVID-19 patients
Source: PLoS One. 2023 Feb 9;18(2):e0276829. doi: 10.1371/journal.pone.0276829 (PMC9910743; doi:10.1371/journal.pone.0276829)
Supplement: S4 Fig — IgG serum responses are shown as percentages of normalized mean signal intensities against 4.8 fmol antigen proteins spike, S2, RBD, HR2 and nucleocapsid. CPT time points for the recipients are shown as one day before the transfusion, one and three days after the transfusion along with corresponding donor’s antibody levels (CP). (PDF) [file pone.0276829.s004.pdf]

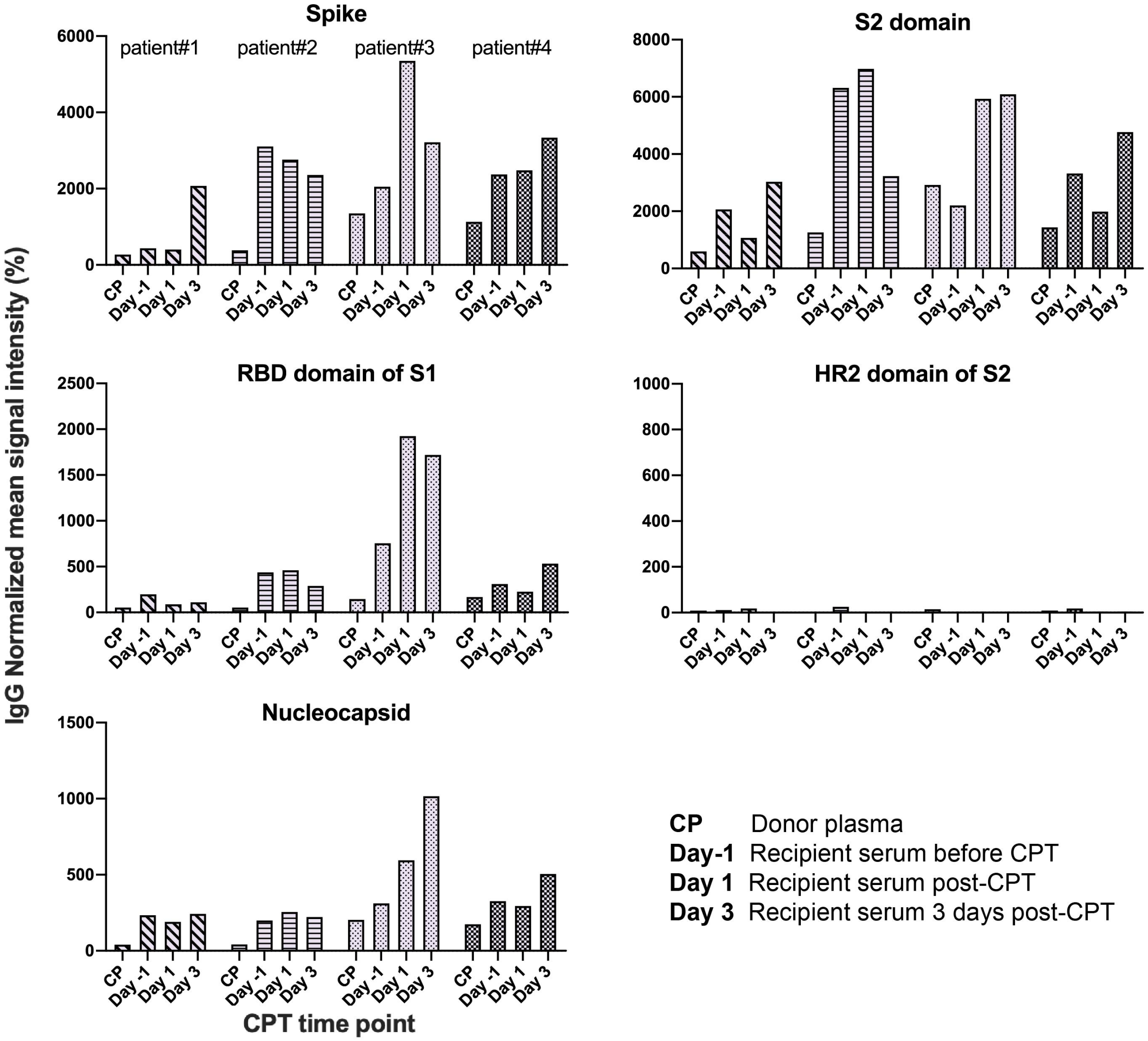

S4 Fig. IgG profiles of four CP therapy donors and four recipients against SARS-CoV-2 proteins before and after CP therapy. IgG serum responses are shown as percentages of normalized mean signal intensities against 4.8 fmol antigen proteins spike, S2, RBD, HR2 and nucleocapsid. CPT time points for the recipients are shown as one day before the transfusion, one and three days after the transfusion along with corresponding donor’s antibody levels (CP).
