# Supplementary material for: SARS-CoV-2 multi-antigen protein microarray for detailed characterization of antibody responses in COVID-19 patients
Source: PLoS One. 2023 Feb 9;18(2):e0276829. doi: 10.1371/journal.pone.0276829 (PMC9910743; doi:10.1371/journal.pone.0276829)
Supplement: S5 Fig — S variants’ shared mutations E484K, N501Y, D614G and Deletion69/70 are shown with their location on S1 domain of S protein. NTD, N-terminal domain; RBD, Receptor binding domain; CTD, C-terminal domain; FP, Fusion peptide; HR1, Heptad repeat 1; HR2, Heptad repeat 2. (PDF) [file pone.0276829.s005.pdf]

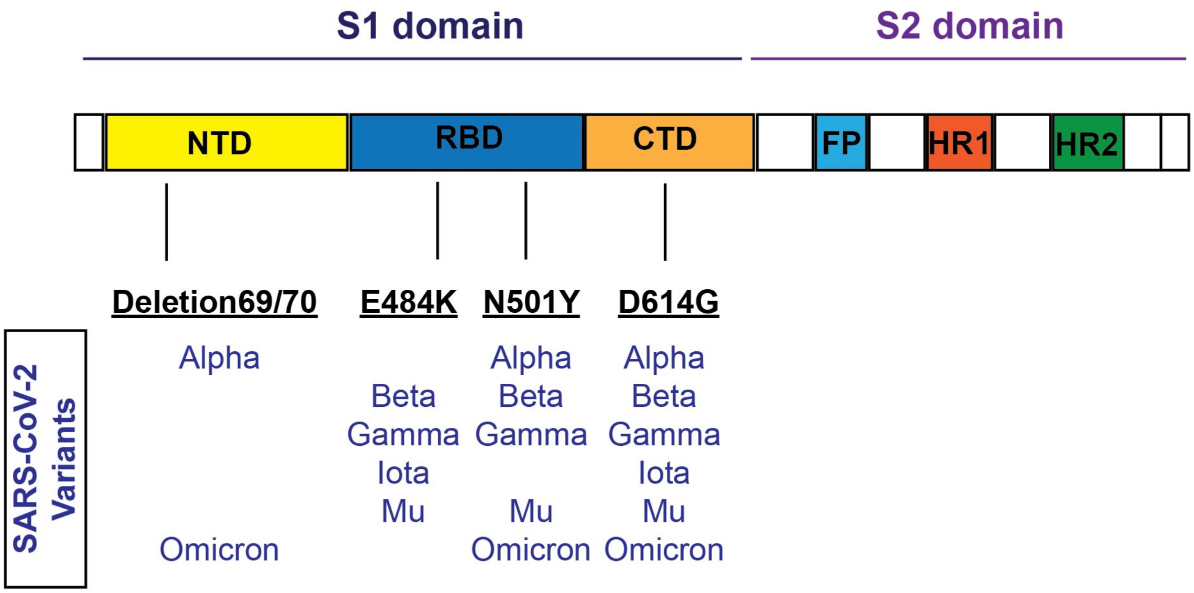

S5 Fig. Shared Spike mutations in SARS-CoV-2 variants. S variants' shared mutations E484K, N501Y, D614G and Deletion69/70 are shown with their location on S1 domain of S protein. NTD, N-terminal domain; RBD, Receptor binding domain; CTD, C-terminal domain; FP, Fusion peptide; HR1, Heptad repeat 1; HR2, Heptad repeat 2.
