# Supplementary material for: SARS-CoV-2 multi-antigen protein microarray for detailed characterization of antibody responses in COVID-19 patients
Source: PLoS One. 2023 Feb 9;18(2):e0276829. doi: 10.1371/journal.pone.0276829 (PMC9910743; doi:10.1371/journal.pone.0276829)

Figure 1. (A) Purified array proteins analyzed by SDS-PAGE under reducing conditions and stained with Coomassie brilliant blue.

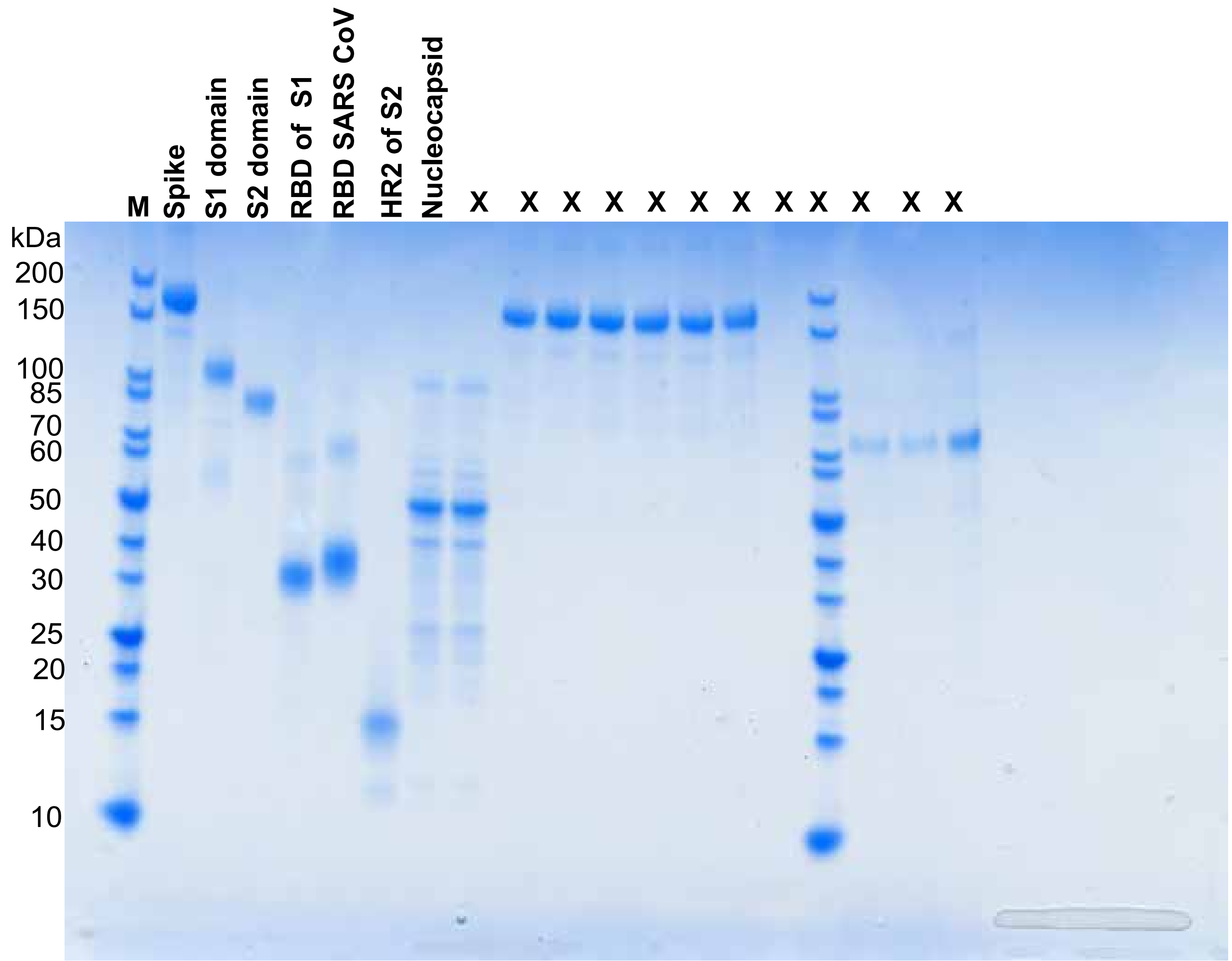

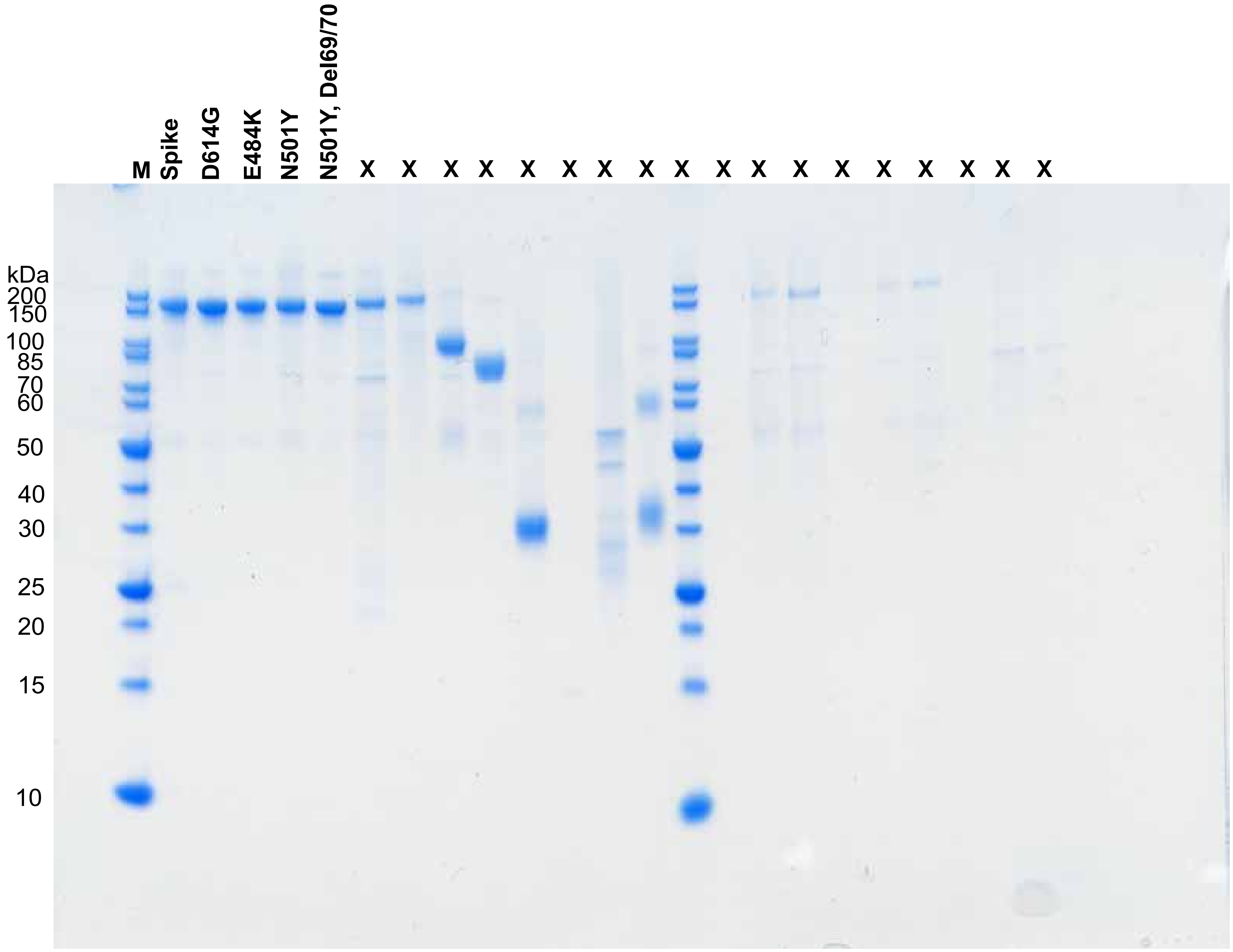

Supplement: S1 Raw images — (PDF) [file pone.0276829.s006.pdf]
